# The prognostic role and metabolic function of GGPS1 in oral squamous cell carcinoma

**DOI:** 10.3389/fmolb.2023.1109403

**Published:** 2023-03-24

**Authors:** Ke Huang, Liang Han, Huimei Xu, Ruiming Xu, Hao Guo, Huihui Wang, Zhaoqing Xu

**Affiliations:** ^1^ Key Laboratory of Preclinical Study for New Drugs of Gansu Province, School of Basic Medical Sciences, Lanzhou University, Lanzhou, China; ^2^ Key Laboratory of Dental Maxillofacial Reconstruction and Biological Intelligence Manufacturing, School of Stomatology, Lanzhou University, Lanzhou, Gansu, China; ^3^ Lanzhou University Second Hospital, Lanzhou University, Lanzhou, Gansu, China; ^4^ The Second Hospital of Dalian Medical University, Dalian, Liaoning, China; ^5^ School of Medicine, Xiamen University, Xiamen, Fujian, China

**Keywords:** GGPS1, prognostic biomarker, tumorigenicity, OSCC, metabolism

## Abstract

**Background:** GGPS1(geranylgeranyl diphosphate synthase 1) is a member of the prenyltransferase family. Abnormal expression of GGPS1 can disrupt the balance between protein farnesylation and geranylgeranylation, thereby affecting a variety of cellular physiologic and pathological processes. However, it is still unknown how this gene could contribute to the prognosis of oral squamous cell carcinoma (OSCC). This study aimed to explore the prognostic role of GGPS1 in OSCC and its relationship with clinical features.

**Methods:** The RNA-seq data and clinical data were obtained from TCGA. The survival analyses, Cox regression analyses, ROC curves, nomograms, calibration curves, and gene function enrichments were established by R software.

**Results:** The results showed that the high expression of GGPS1 in OSCC is related to poor prognosis. At the same time, multivariate Cox regression analyses showed that GGPS1 could be an independent prognostic biomarker, and its gene expression level is closely related to the histological stage of cancer. GGPS1 may promote tumorigenesis because of its metabolic function.

**Conclusion:** This study came to a conclusion that GGPS1, whose high expression has a significantly unfavorable meaning toward the prognosis of OSCC, can act as a novel independent biomarker for OSCC.

## 1 Introduction

Oral cancer is one of the most common malignant tumors in the world (Sung et al., 2021). Oral squamous cell carcinoma (OSCC) derives from the oral squamous epithelium and is the most common oral cancer with high morbidity and mortality. The early diagnosis of OSCC is relatively easy, but presentation with advanced disease is not uncommon (Montero and Patel, 2015; Bugshan and Farooq, 2020; Johnson et al., 2020; Vitório et al., 2020; Chamoli et al., 2021). The treatments are single, such as surgery or chemotherapy. At present, relatively new therapeutic research fields are immunization and molecular targeted therapy, but it is a long distance before being put into clinical application. Therefore, it is very important to improve and exploit the tools of early diagnosis for the better prevention and treatment of oral cancer. There is an urgent need for biomarkers of OSCC to act as indicators of diagnosis and prognosis and may provide ideas for treatment options on the molecular scale, further better specific treatment and survival rate rates.

GGPS1 (geranylgeranyl diphosphate synthase 1) is a member of the prenyltransferase family and encodes a protein with geranylgeranyl diphosphate (GGPP) synthase activity. Abnormal expression of GGPS1 can disrupt the balance between protein farnesylation and geranylgeranylation, and result in a variety of cellular physiologic and pathological processes, thereby affecting inflammation and organ damage (Xu et al., 2015). The researches of GGPS1 on the functions and the relationship between diseases and GGPS1 involve an extremely extensive range. GGPS1-knockout plays a part in restraining ventilator-induced lung injury and attenuating sepsis-induced acute lung injury (Li et al., 2021; Wang et al., 2022). In Perrault syndrome and A typical femur fractures, GGPS1 may be one of the genetic factors (Foley et al., 2020; Zhou et al., 2021; Faridi et al., 2022). GGPS1 deficiency could result in dystocia by disrupting uterine contraction (Sang et al., 2021). In liver diseases, GGPS1 is critical for modulating acute obstructive cholestatic liver injury and can serve as a biomarker in Hepatocellular carcinoma (Yu et al., 2014; Jia et al., 2020). In lung adenocarcinoma, the overexpression of GGPS1 contributes to tumor metastasis and is correlated with poor prognosis (Wang et al., 2018). Besides, it has been proved that the expression of GGPS1 participated in and affected the treatment of two anticancer drugs for OSCC (Xu et al., 2021). But the concrete characters of GGPS1 in OSCC remain unclear.

This study obtained RNA-seq data and clinical data from TCGA to identify the associations between GGPS1 and OSCC, concluding that GGPS1 expression was higher in tumor tissues and the high expression could portend a worse prognosis for OSCC. Next, several bioinformatic analyses such as Cox regression analysis, ROC curves, nomogram, and calibration curves, assessed the diagnostic value of GGPS1 for OSCC and made it clear that GGPS1was an independent predictor of poor prognosis. Gene function analyses indicated that GGPS1 expression was significantly related to Terpenoid backbone biosynthesis, regulation of small molecule metabolic process, regulation of lipid metabolic process, and cholesterol metabolic process. All in all, this study aimed to better understand the prognosis of oral squamous cell carcinoma related to GGPS1 expression and the function of the GGPS1, so as to broaden the scope of diagnostic and prognostic indicators for OSCC.

## 2 Materials and methods

### 2.1 Data collection and integration

The dataset consists of 361 samples (329 tumor tissues and 32 adjacent non-cancerous tissues from TCGA) (Blum et al., 2018). The RNA-seq data and corresponding clinical information were downloaded from TCGA, and RNA-seq data in TPM format standardized by the Toil process was downloaded from UCSC XENA (https://xenabrowser.net/datapages/) (Vivian et al., 2017). To further compare the mRNA expression level between tumor and non-tumor samples, the logarithm of 2-fold change (log2FC) was calculated. Clinical information of the OSCC patients included TMN stage, clinical stage, histologic grade, Overall Survival (OS) event, Disease Specific Survival (DSS) event, Progression Free Interval (PFI) event, etc. Samples with unclear or error information were excluded. For GGPS1 correlation analysis, the RNA-seq data from the TCGA (https://portal.gdc.cancer.gov/) in the HTSeq-FPKM format were converted to TPM format, and log2FC is converted. The significant correlation condition was |cor_spearman|≥0.4 and *p* _ spearman <0.05. Data without corresponding clinical information were excluded. Specifically, we included samples from specific oral cancer sites, including the alveolar ridge, base of tongue, buccal mucosa, floor of mouth, hard palate, oral cavity, and oral tongue, and excluded samples from non-oral cancer sites such as the hypopharynx, larynx, lip, oropharynx, and tonsil. STRING database (https://string-db.org/) aims to integrate all known and predicted associations between proteins and analyze their sources, thus, it was used to explore the protein-protein interaction (PPI) networks of GGPS1 (Szklarczyk et al., 2021). In addition, GeneMANIA (http://www.genemania.org), a web interface that identifies functionally similar genes using available genomics and proteomics data, was used for the analyses of the protein-protein interaction network of GGPS1 (Warde-Farley et al., 2010). For single gene difference analysis, the RNA-seq data from the TCGA (https://portal.gdc.cancer.gov/) in the HTSeq-Counts format were converted to log2FC (Love et al., 2014). The conditions of single gene difference analysis set as Log2FC > 2 and *p*-value <0.05. MSigDB (http://www.broadinstitute.org/msigdb) was used to perform gene set enrichment analysis(GSEA) (Liberzon et al., 2011; Liberzon et al., 2015).

### 2.2 Databases exploration

The HPA database (https://www.proteinatlas.org) is an extensive project that aims to map the entire human proteome by utilizing a combination of antibody-based proteomics and other omics techniques. In this study, the protein expression of GGPS1 was explored using the HPA database, and immunohistochemical samples of oral mucosa from patient ID 3917 and head and neck squamous cell carcinoma (HNSC) from patient ID 3669 were extracted.

The TISDB (http://cis.hku.hk/TISIDB/) database is a web-based platform that integrates multiple resources related to tumor immunology. In our study, we utilized the TISDB database for analyzing drugs that target GGPS1. This database offers a user-friendly interface and provides access to diverse data resources related to tumor immunology.

### 2.3 Survival and statistical analyses

Patients with OSCC were divided into high and low GGPS1 expression groups according to the median expression level. Kaplan-Meier (KM) survival analyses were used to evaluate the association between GGPS1 expression level and Overall Survival (OS), Disease Specific Survival (DSS), and Progression Free Survival (PFS) by the R package (survminer, version 0.4.9 and survival, version 3.2.10). Calculated hazard ratios (HR) were presented, and *p* < 0.05 was statistically significant.

### 2.4 Univariate and multivariate cox regression analyses

The role of univariate and multivariate Cox regression was assessing whether gender, age, TNM stage, the expression of GGPS1and so on were independent prognostic factors for the survival of OSCC patients. This study calculated hazard ratios (HR) and 95% confidence intervals (CI), and the significance threshold was set at *p* < 0.05. R package (survival, version 3.2.10) was a procedure of data processing. HR > 1 indicates disadvantageous factors.

### 2.5 Construction of nomograms, calibration plots, and ROC curves

The nomogram was used to make a forecast for OSCC prognosis by predicting the 1-, 3- and 5-year survival probability. Calibration curves were drawn to show the prediction effect. The ROC curves with time dependence, by using the R package (pROC, version 1.17.0.1 and ggplot2, version 3.3.3), were obtained for assessing the diagnostic value of GGPS1 mRNA expression.

### 2.6 GGPS1-related function enrichment analyses

This study used the R package (DESeq2, version 1.26.0) to analyze Different Expressed Genes (Love et al., 2014). Based on the dataset of heatmaps, STRING database, and GeneMANIA, there were Gene Ontology (GO) and Kyoto Encyclopedia of Genes and Genomes (KEGG) analyses to evaluate GGPS1-associated potential gene functions with the R package (org.Hs.e.g.,.db, version 3.10.0 and clusterProfiler, version 3.14.3) (Yu et al., 2012). Gene Set Enrichment Analysis (GSEA) for interpreting gene expression data assesses whether an *a priori* defined set of genes shows statistically significant, concordant differences between two biological states (Subramanian et al., 2005; Subramanian et al., 2007). Set the False Discovery Rate (FDR) < 0.25 and the p.adjust<0.05 condition to be significant enrichment. The computational method was carried out using the R package clusterProfiler (version 3.14.3) and the R package ggplot2 (version 3.3.3) for data visualization.

### 2.7 Statistical analyses

This study conducted statistical calculations and graphing by R software (version 3.6.3, R Core Team, Vienna, Austria) and Adobe Photoshop software (version 25.0.0.60, Adobe Inc., Mountain View, CA, United States). The correlation analysis between clinical information and gene expression was implemented by Cox regression. Same as in previous studies, *p* < 0.05 was set up as the cut-off criterion.

## 3 Results

### 3.1 Clinical characteristics of the OSCC patients

The clinical and gene expression data of 329 tumor tissues and 32 adjacent non-cancerous tissues were downloaded from TCGA. This part was grouped according to the clinical information of OSCC, such as TMN stage, clinical stage, gender, race, age, and so on. Samples with unclear or error information were excluded, *p* < 0.05 suggested that this clinical feature may be related to GGPS1 expression in terms of statistical significance (Table 1). The results showed that Primary therapy outcome, histologic grade, Overall Survival (OS) event, Disease Specific Survival (DSS) event, and Progression Free Interval (PFI) event may be related to GGPS1 expression.

**TABLE 1:** The GGPS1 expressions in OSCC patients with different clinical information.

Characteristic	Levels	Low expression of GGPS1	High expression of GGPS1	*p*
*n*		164	165	
T stage, *n* (%)	T1	11 (3.4%)	7 (2.2%)	0.698
	T2	53 (16.6%)	52 (16.3%)	
	T3	41 (12.9%)	41 (12.9%)	
	T4	53 (16.6%)	61 (19.1%)	
N stage, *n* (%)	N0	89 (28.3%)	79 (25.1%)	0.371
	N1	27 (8.6%)	29 (9.2%)	
	N2	37 (11.7%)	51 (16.2%)	
	N3	2 (0.6%)	1 (0.3%)	
M stage, *n* (%)	M0	154 (49.4%)	156 (50%)	0.498
	M1	2 (0.6%)	0 (0%)	
Clinical stage, *n* (%)	Stage I	8 (2.5%)	3 (0.9%)	0.340
	Stage II	38 (11.9%)	41 (12.9%)	
	Stage III	35 (11%)	30 (9.4%)	
	Stage IV	77 (24.1%)	87 (27.3%)	
Radiation therapy, *n* (%)	No	54 (18.3%)	62 (21%)	0.245
	Yes	97 (32.9%)	82 (27.8%)	
Primary therapy outcome, *n* (%)	PD	11 (4%)	24 (8.6%)	0.045
	SD	2 (0.7%)	2 (0.7%)	
	PR	1 (0.4%)	2 (0.7%)	
	CR	128 (46%)	108 (38.8%)	
Gender, *n* (%)	Female	57 (17.3%)	45 (13.7%)	0.178
	Male	107 (32.5%)	120 (36.5%)	
Race, *n* (%)	Asian	4 (1.3%)	5 (1.6%)	0.099
	Black or African American	6 (1.9%)	15 (4.7%)	
	White	150 (47.2%)	138 (43.4%)	
Age, *n* (%)	≤60	78 (23.8%)	77 (23.5%)	1.000
	>60	86 (26.2%)	87 (26.5%)	
Histologic grade, *n* (%)	G1	36 (11.2%)	16 (5%)	0.009
	G2	99 (30.8%)	101 (31.5%)	
	G3	27 (8.4%)	40 (12.5%)	
	G4	1 (0.3%)	1 (0.3%)	
Anatomic neoplasm subdivision, *n* (%)	Alveolar Ridge	11 (3.3%)	7 (2.1%)	0.731
	Base of tongue	12 (3.6%)	11 (3.3%)	
	Buccal Mucosa	9 (2.7%)	13 (4%)	
	Floor of mouth	32 (9.7%)	29 (8.8%)	
	Hard Palate	5 (1.5%)	2 (0.6%)	
	Oral Cavity	36 (10.9%)	36 (10.9%)	
	Oral Tongue	59 (17.9%)	67 (20.4%)	
Smoker, *n* (%)	No	45 (13.9%)	42 (13%)	0.828
	Yes	117 (36.2%)	119 (36.8%)	
Alcohol history, *n* (%)	No	58 (18.1%)	47 (14.6%)	0.191
	Yes	101 (31.5%)	115 (35.8%)	
Lymphovascular invasion, *n* (%)	No	89 (37.2%)	75 (31.4%)	0.056
	Yes	30 (12.6%)	45 (18.8%)	
Lymphnode neck dissection, *n* (%)	No	22 (6.7%)	23 (7%)	1.000
	Yes	141 (43.1%)	141 (43.1%)	
OS event, *n* (%)	Alive	106 (32.2%)	73 (22.2%)	<0.001
	Dead	58 (17.6%)	92 (28%)	
DSS event, *n* (%)	Alive	120 (38.5%)	99 (31.7%)	0.021
	Dead	37 (11.9%)	56 (17.9%)	
PFI event, *n* (%)	Alive	106 (32.2%)	88 (26.7%)	0.049
	Dead	58 (17.6%)	77 (23.4%)	
Age, median (IQR)		61 (53.75, 71)	61 (54, 69.25)	0.847

### 3.2 Gene expressions of GGPS1 in OSCC

**FIGURE 1:**
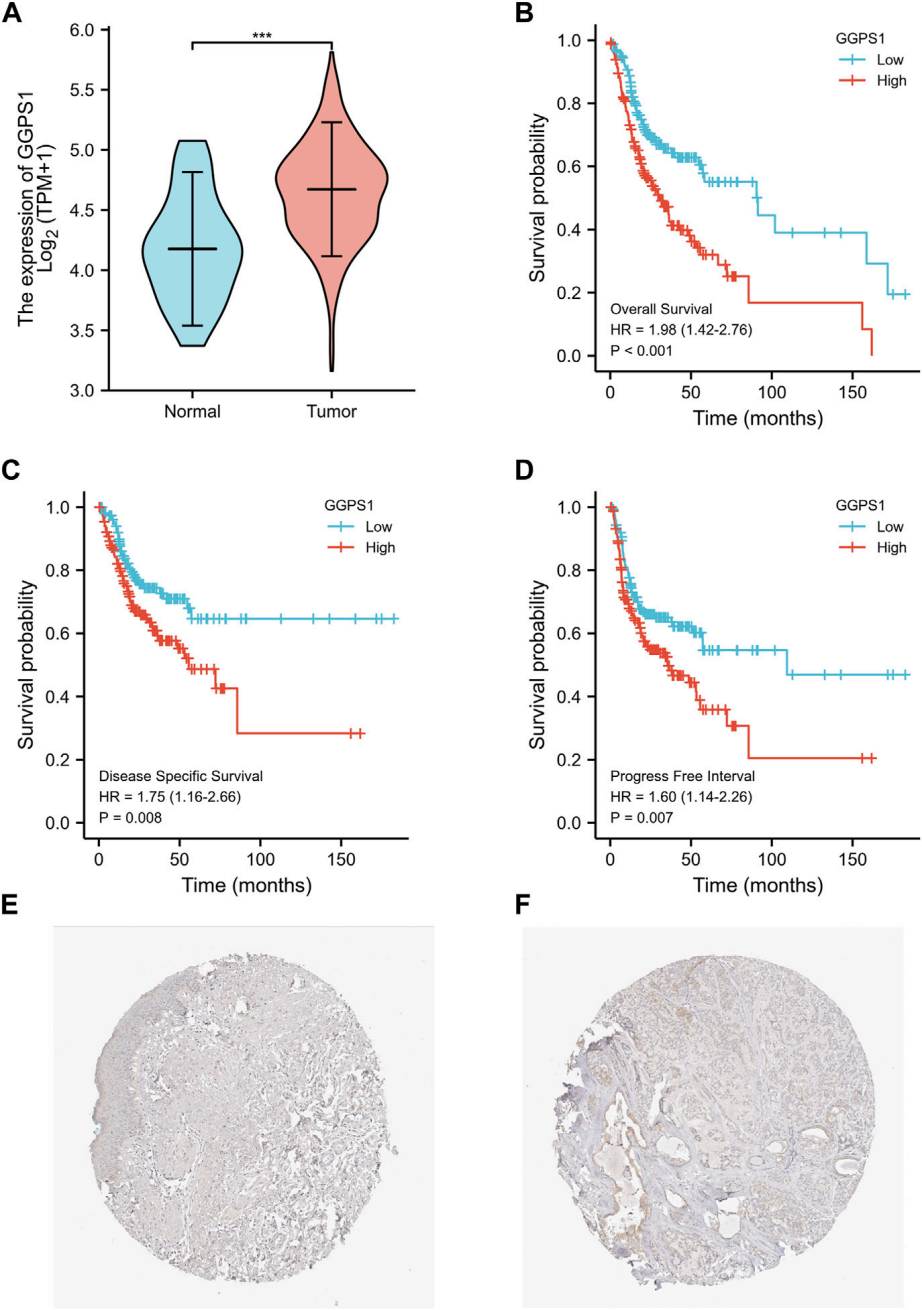
The GGPS1 expressions and survival analysis in OSCC. **(A)** The differential expression analysis of GGPS1 in patients with OSCC. **(B)** KM curves of the associations between GGPS1 expressions and overall survival. **(C)** KM curves of the associations between GGPS1 expressions and disease specific survival. **(D)** KM curves of the associations between GGPS1 expressions and progress free interval. (***, *p* < 0.001). **(E)** Immunohistochemistry staining image of GGPS1 in oral mucosa tissue obtained from the HPA database. **(F)** Immunohistochemistry staining image of GGPS1 in HNSC obtained from the HPA database.

To begin with, the useful data about expressions of GGPS1 were analyzed and the results showed that the expressions of GGPS1 in tumor tissues were significantly higher in normal tissues (Figure 1A). The data of tumor samples were divided into two parts with a line of demarcation that was derived from the median expression level of GGPS1. Next, KM survival analyses were performed to assess the connection between GGPS1 expression levels and Overall Survival (OS) events (Figure 1B), Disease Specific Survival (DSS) events (Figure 1C), and Progression Free Interval (PFI) events (Figure 1D). It is worth noting that the GGPS1 expressions were very closely linked to OS, DSS, and PFI of the patients of OSCC. In addition, we utilized the HPA database to investigate the expression of GGPS1 in normal oral mucosa and HNSC tissue. Our findings indicated that GGPS1 protein expression was not significantly detected in oral mucosal tissues, whereas it was detected in HNSC (Figures 1E, F). The above results taken together indicated that GGPS1 possibly impacts cancer and the high expression level not only shortened the Progression Free Interval but also presaged a worse prognosis.

### 3.3 Correlations between clinical characteristics and expressions of GGPS1 in OSCC

**FIGURE 2:**
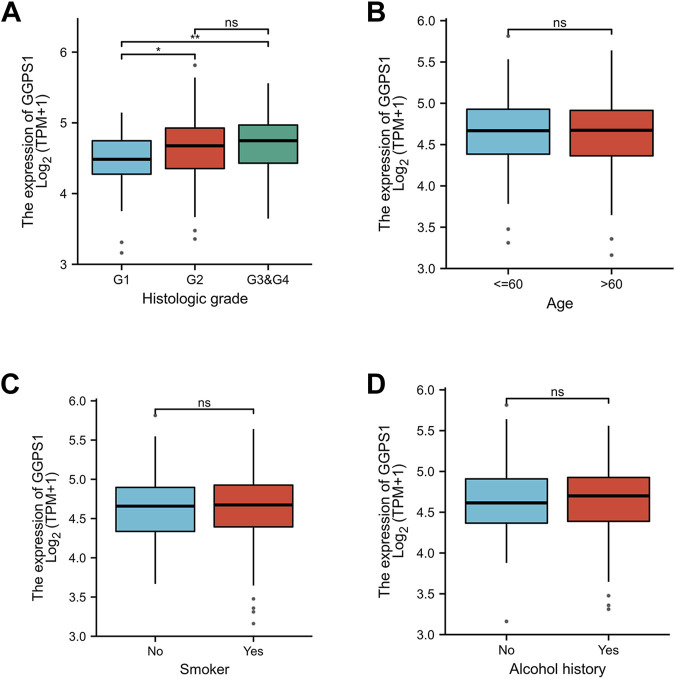
The associations between expressions of GGPS1 and clinical characteristics. **(A)** Histological grade. **(B)** Age. **(C)** Smoker. **(D)** Alcohol history. (ns, no statistically difference; *, *p* < 0.05; **, *p* < 0.01)

Kruskal–Wallis Test concerning clinical characteristics of OSCC and expressions of GGPS1 were used to analyze the correlations between the two. First of all, data analyses of the expressions of GGPS1 in histologic grade showed that with the deterioration of histological stages (Figure 2A), the expression increased obviously, and it was statistically significant. Interestingly, through the statistics and analysis of the data, it was found that the expression levels of GGPS1 were irrelevant to age (Figure 2B), smoker (Figure 2C), and alcohol history (Figure 2D). Results showed that a higher expression level of GGPS1 predicted a worse prognosis.

### 3.4 Diagnostic value of GGPS1 in OSCC

**FIGURE 3:**
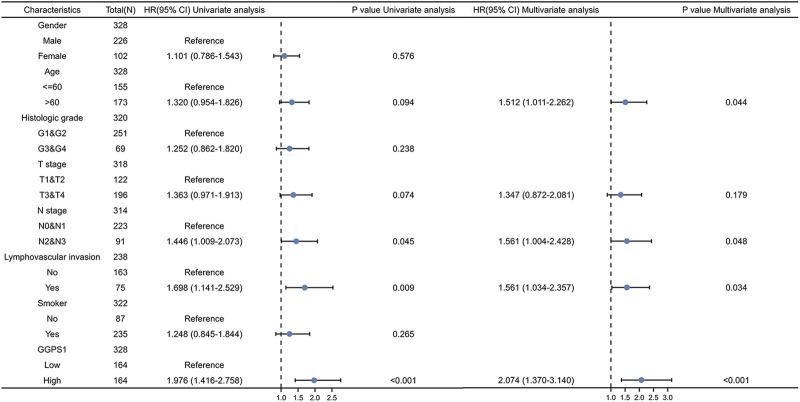
Univariate and multivariate Cox regression analysis. HR > 1 indicates disadvantageous factors.

**FIGURE 4:**
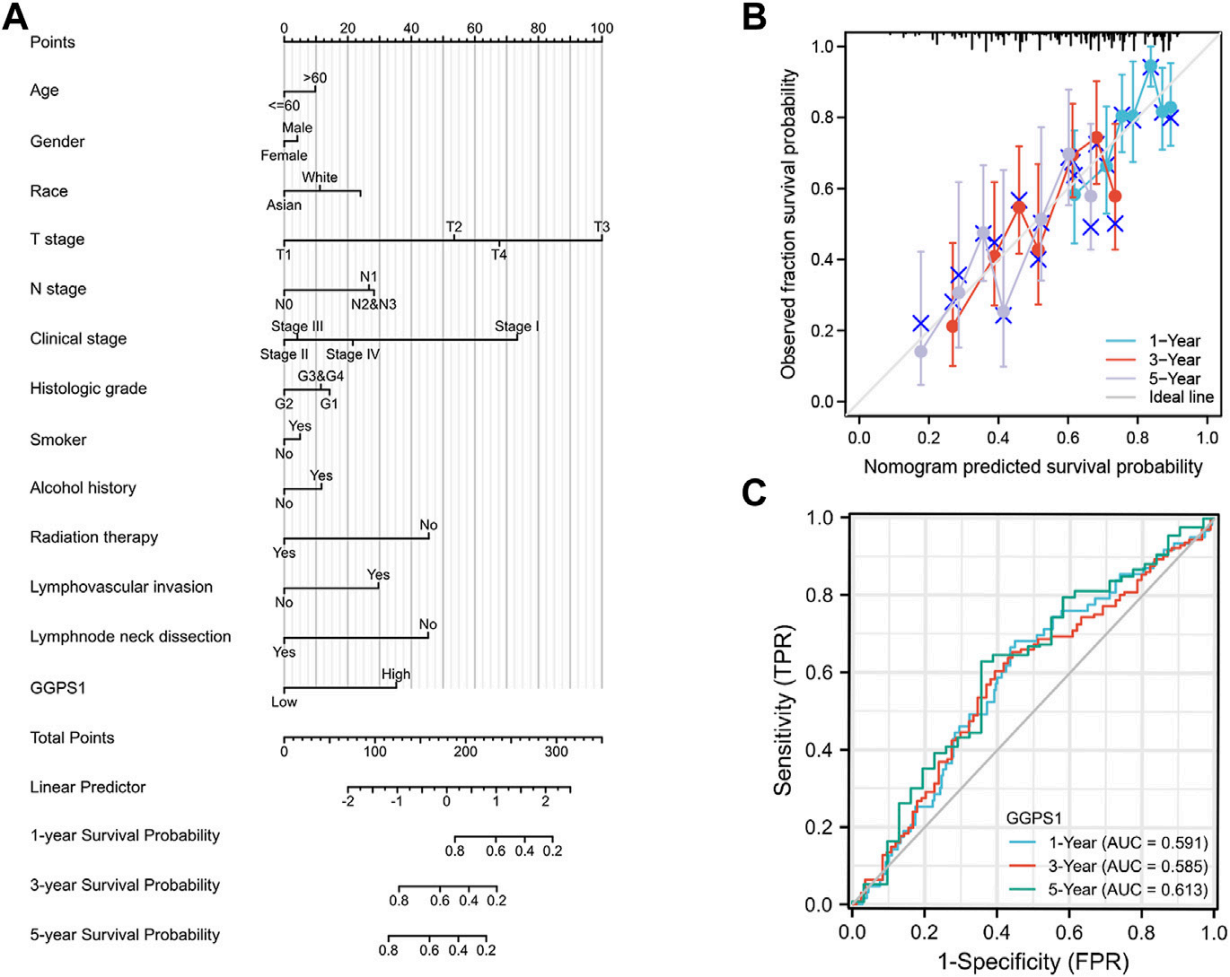
Diagnostic value of GGPS1 in OSCC. **(A)** The nomogram combining GGPS1 expression with key clinical characteristics. **(B)** The calibration plot of the nomogram for predicting 1-,3-and 5-year overall survival probability. **(C)** The diagnostic value of GGPS1 mRNA expression.

The role of univariate and multivariate Cox regression was to assess whether the expression of GGPS1 and clinical information of OSCC such as gender, age, histologic grade, and lymphovascular invasion were independent prognostic factors for the survival of patients. There were results that N stage, lymphovascular invasion, and most importantly, the expression of GGPS1 were risk factors. According to the multivariate analysis, these three factors could be regarded as independent prognostic factors as well (Figure 3). The nomogram was used to make a forecast for OSCC prognosis by predicting the 1-, 3- and 5-year survival probability (Figure 4A). The calibration plots of the above nomogram-predicated survival probability were very close to the ideal reference line (Figure 4B). Furthermore, the ROC curves with time dependence, drafted to assess the diagnostic value of GGPS1 mRNA expression, showed that the above predictions are reliable (Figure 4C). The results revealed that GGPS1was an independent predictor of poor prognosis with excellent diagnostic value.

### 3.5 GGPS1 correlation analysis and GGPS1 gene function prediction

**FIGURE 5:**
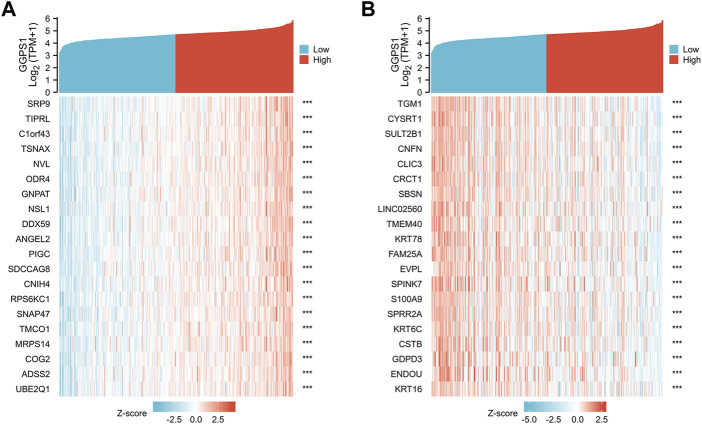
**(A)** The top 20 genes positively correlated with GGPS1. **(B)** The top 20 genes negatively correlated with GGPS1.

To further understand the gene functions of GGPS1, analyses of gene correlations were performed. According to the analysis results, there were 23,818 genes positively correlated with GGPS1, and in parallel, 1,121 genes negatively correlated (Supplementary Table 1). The top 20 genes positively or negatively correlated with GGPS1 were shown in heat maps (Figures 5A, B).

**FIGURE 6:**
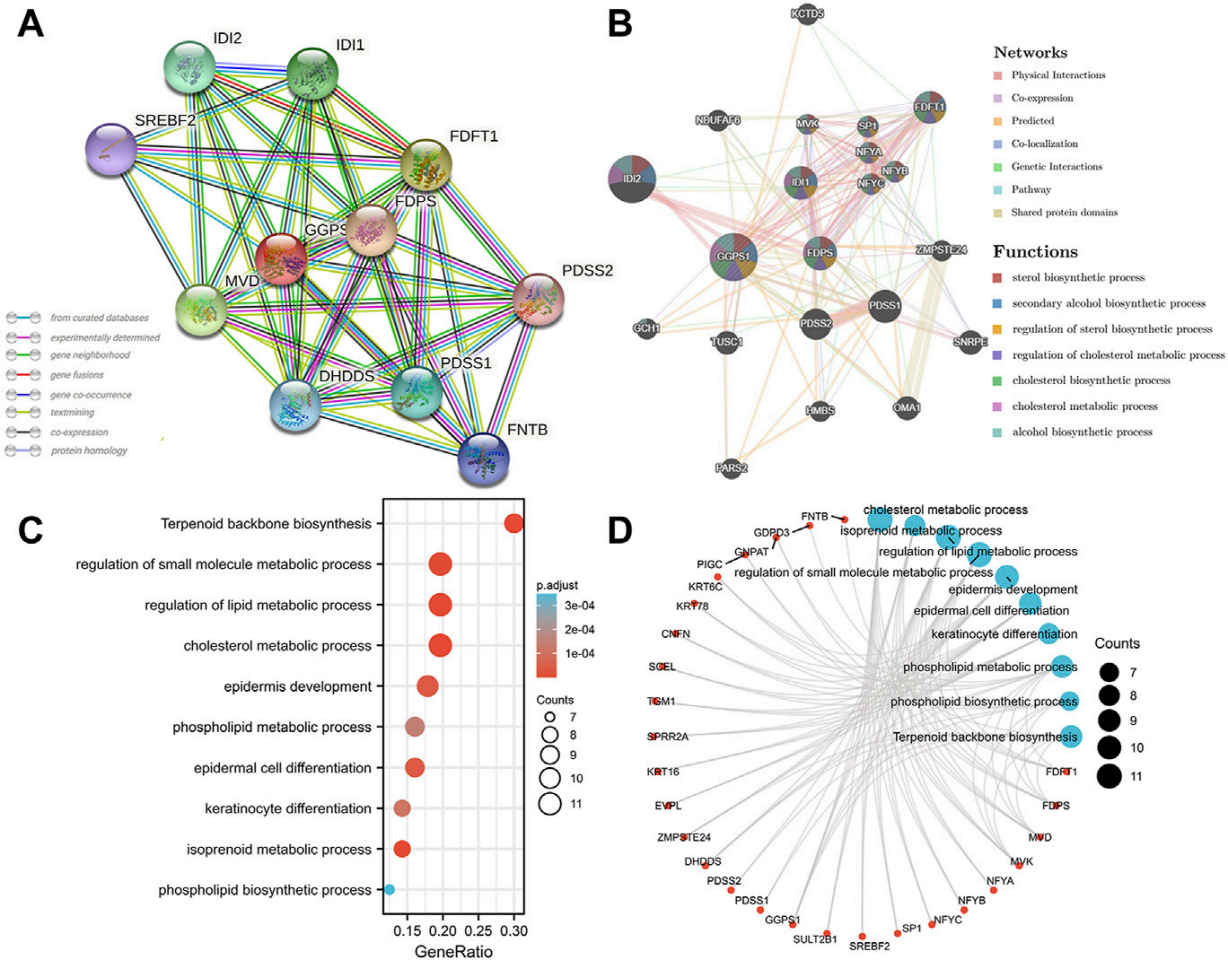
Protein-protein interaction (PPI) network and functional enrichment of GGPS1. **(A)** PPI network analysis by the STRING database. **(B)** PPI network analysis by the GeneMANIA database. **(C)** Bubble plot of GO/KEGG enrichment analysis. **(D)** Network visualization of GO/KEGG enrichment analysis.

After obtaining representative genes correlated with GGPS1, the protein-protein interaction (PPI) network analysis was mapped. It was found that there were 10 genes associated with GGPS1 using the STRING database (Figure 6A), and 20 genes related to GGPS1 using the GeneMANIA database (Figure 6B). Results indicated that some genes such as IDI2, IDI1, FDPS, and FDFT1 related to cholesterol synthesis were most closely related to GGPS1 (Nakamura et al., 2015; Dong et al., 2020; Ershov et al., 2021). In addition, to explore the potential biological functions related to GGPS1 expression, GO/KEGG analysis was executed and outcomes showed that GGPS1 expressions are associated with a series of functions such as Terpenoid backbone biosynthesis, regulation of small molecule metabolic process, regulation of lipid metabolic process, cholesterol metabolic process, and so on (Supplementary Table 2, Figures 6C, D).

### 3.6 Single gene difference analysis and GSEA enrichment analysis

**FIGURE 7:**
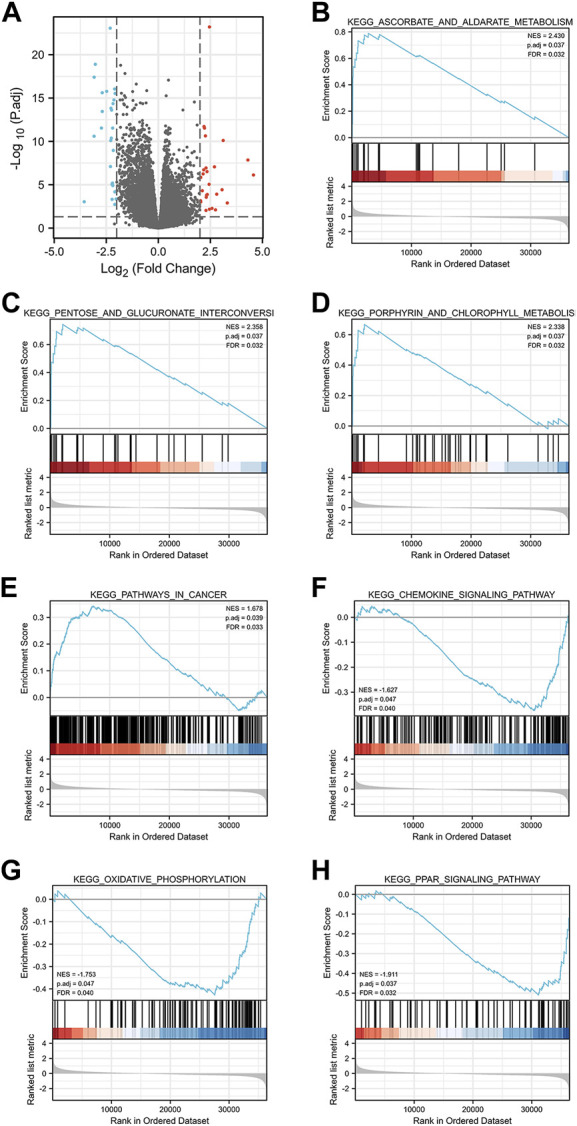
**(A)** Volcano plot of GGPS1. **(B–H)** GSEA analysis of GGPS1.

Single gene difference analysis of GGPS1 was performed and the corresponding volcano map was drawn (Supplementary Table 3, Figure 7A). The total number excluding the null value is 35,620. Selected threshold value is |log2(FC)|>2 & p.adj<0.05, and the number meeting this threshold value is 54, among which 27 are high expressions (log2FC is positive) and 27 are low expressions (log2FC is negative). Furthermore, Gene Set Enrichment Analysis (GSEA) analysis was carried out for GGPS1 gene function prediction (Supplementary Table 4). Outcomes of GSEA analyses showed that a high expression of GGPS1 was associated with the activation of ascorbate and aldarate metabolism (Figure 7B), pentose and glucuronate interconversions (Figure 7C), porphyrin and chlorophyll metabolism (Figure 7D), pathways in cancer (Figure 7E), chemokine signaling pathway (Figure 7F), oxidative phosphorylation (Figure 7G), and PPAR signaling pathway (Figure 7H). The above-mentioned results comprehen show that GGPS1 did play a role in the development of OSCC.

### 3.7 Drugs targeting GGPS1

**FIGURE 8:**
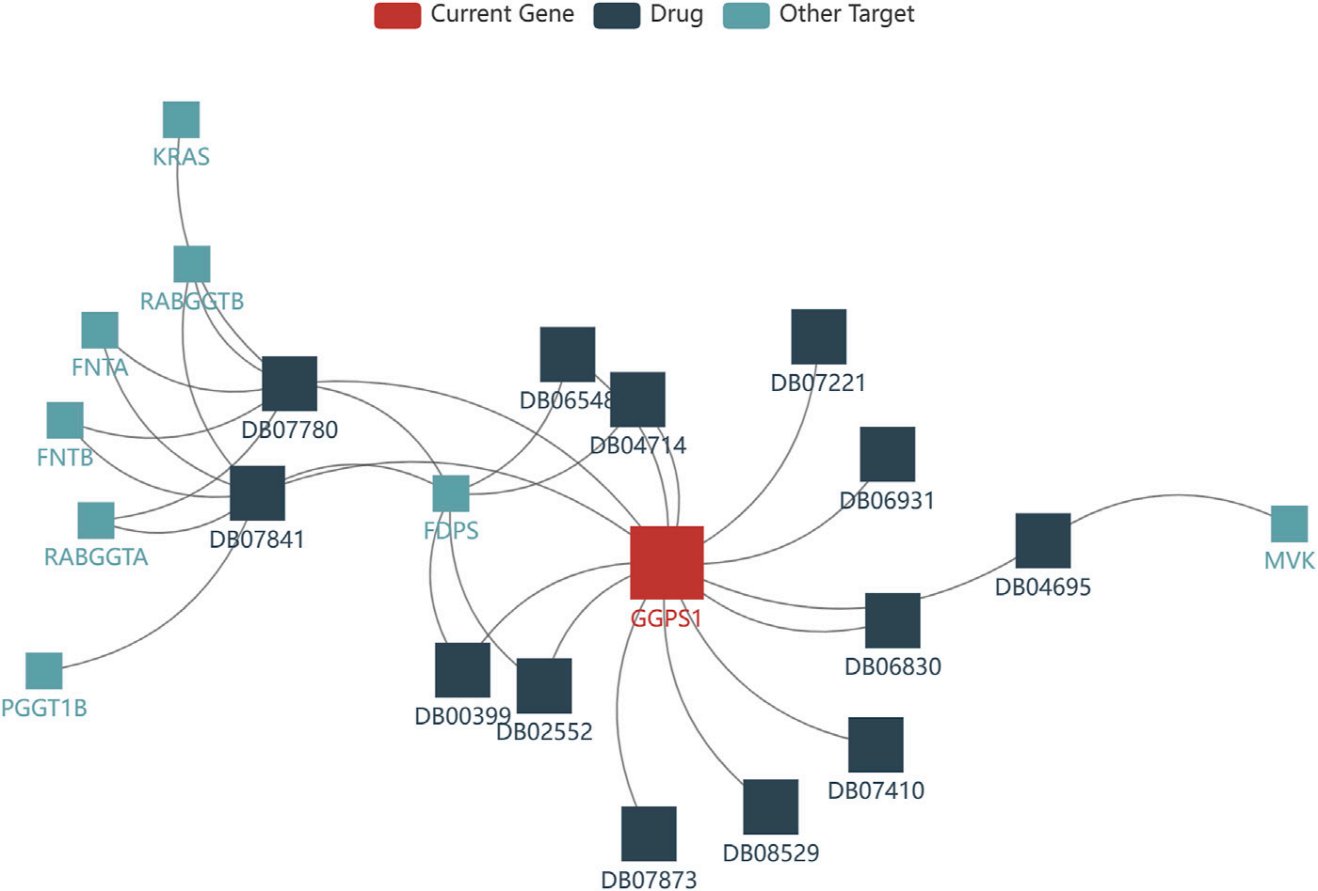
Drugs targeting GGPS1 identified from the TISDB database.

The development of drugs targeting GGPS1 is crucial for the effective treatment of cancer patients. In this study, we utilized the TISDB database to analyze the available drugs that target GGPS1, and identified a total of 14 small molecule drugs. Notably, farnesyl diphosphate (DB07780) and geranylgeranyl diphosphate (DB07841) exhibited the highest number of targets, with a total of 7 targets (Figure 8). Additional information can be found in Table 2.

**TABLE 2:** Details on Drugs targeting GGPS1.

ID	Name	Drug type	Targets	Targets
DB00399	Zoledronic acid	Small Molecule	FDPS, GGPS1	2
DB02552	Geranyl Diphosphate	Small Molecule	FDPS, GGPS1	2
DB04695	FARNESYL THIOPYROPHOSPHATE	Small Molecule	GGPS1, MVK	2
DB04714	ISOPENTENYL PYROPHOSPHATE	Small Molecule	FDPS, GGPS1	2
DB06548	Minodronic acid	Small Molecule	FDPS, GGPS1	2
DB06830	(1-HYDROXYHEPTANE-1,1-DIYL)BIS(PHOSPHONIC ACID)	Small Molecule	GGPS1	1
DB06931	(1-HYDROXYNONANE-1,1-DIYL)BIS(PHOSPHONIC ACID)	Small Molecule	GGPS1	1
DB07221	(2,2-DIPHOSPHONOETHYL)(DODECYL)DIMETHYLPHOSPHONIUM	Small Molecule	GGPS1	1
DB07410	[2-(3-DIBENZOFURAN-4-YL-PHENYL)-1-HYDROXY-1-PHOSPHONO-ETHYL]-PHOSPHONIC ACID	Small Molecule	GGPS1	1
DB07780	FARNESYL DIPHOSPHATE	Small Molecule	FDPS, FNTA, FNTB, GGPS1, KRAS, RABGGTA, RABGGTB	7
DB07841	Geranylgeranyl diphosphate	Small Molecule	FDPS, FNTA, FNTB, GGPS1, PGGT1B, RABGGTA, RABGGTB	7
DB07873	(1-HYDROXYDODECANE-1,1-DIYL)BIS(PHOSPHONIC ACID)	Small Molecule	GGPS1	1
DB08529	(6E,11E)-HEPTADECA-6,11-DIENE-9,9-DIYLBIS(PHOSPHONIC ACID)	Small Molecule	GGPS1	1
DB07841	Geranylgeranyl diphosphate	Small Molecule	FDPS, FNTA, FNTB, GGPS1, PGGT1B, RABGGTA, RABGGTB	7
DB07873	(1-HYDROXYDODECANE-1,1-DIYL)BIS(PHOSPHONIC ACID)	Small Molecule	GGPS1	1
DB08529	(6E,11E)-HEPTADECA-6,11-DIENE-9,9-DIYLBIS(PHOSPHONIC ACID)	Small Molecule	GGPS1	1

## 4 Discussion

Cancers of the oral cavity are the most common non-melanoma head and neck cancer in the world with delayed clinical detection, poor prognosis, without specific biomarkers for the disease, and expensive therapeutic alternatives (Rivera, 2015; Howard et al., 2021). In oral cavity malignancies, approximately 90% are squamous cell carcinoma (OSCC) (Ellington et al., 2020). As an aggressive tumor, the prognosis of OSCC has exhibited little improvement in the last three decades (Sasahira and Kirita, 2018). Therefore, it is critically important to detect and make an early referral of premalignant lesions and oral cancers and ongoing surveillance (Wong and Wiesenfeld, 2018). It would be advisable that we pay close attention to the identification of new biomarkers characterizing OSCC.

Firstly, this study classified the GGPS1 expressions in OSCC patients with different clinical data. The included factors were of great significance in the occurrence, development, and epidemiology of OSCC. For example, tobacco and alcohol exposure is one of the main risk factors, tumor staging gives information about loco-regional and metastatic spread and patient management is usually based on the TNM stage or clinical stage (Paré and Joly, 2017; Panarese et al., 2019). The samples related to gender, race, and age are also an important part of OSCC epidemiology. Meanwhile, treatment of OSCC mainly includes surgery, radiation therapy, and chemotherapy (de Souza et al., 2020; Nandini et al., 2020). Survival outcomes after treatment are also used to analyze prognostic indicators (Zanoni et al., 2019). Then, the clinical information was statistically analyzed. Based on the *p*-value < 0.05, we have a preliminary understanding of the risk factors related to GGPS1 such as primary therapy outcome, histologic grade, Overall Survival (OS) event, Disease Specific Survival (DSS) event, Progression Free Interval (PFI) event may be related to GGPS1 expression.

Expressions of GGPS1 were analyzed and the result showed that the expressions of GGPS1 in tumor tissues were significantly higher in normal tissues. Subgroup expression difference showed that the more deteriorative histological stages, the more increased expression. Interestingly, the expression levels of GGPS1 seemed irrelevant to age, smoker, and alcohol history. In fact, as early as 1995, the relationship between smoking and oral cancer has been established (Gupta et al., 2008). The most significant pathology comes from chemical and molecular interactions with tobacco-related products like several genetic alterations and non-specific global hypomethylation (Gasche and Goel, 2012; Ford and Rich, 2021). Global DNA hypomethylation is a common feature of oral cancers (Foy et al., 2015). The role of alcohol as an independent factor in oral carcinogenesis is still unclear but there is synergy with tobacco in the development of oral cancer (Mello et al., 2019). GGPS1 can modulate protein prenylation that is required for protein membrane-anchoring and activation, mainly related to metabolism (Jia et al., 2016). Different mechanisms of action may be a reason for the expression levels of GGPS1 irrelevant to smoker and alcohol. But all in all, results showed that a higher expression level of GGPS1 predicted a worse prognosis. Subsequently, univariate and multivariate Cox regression, nomograms, calibration curves, and ROC curves showed that GGPS1 could serve as an independent prognostic factor of the OSCC.

The PPI network of GGPS1 was explored. Results indicated that some genes such as IDI2, IDI1, FDPS, and FDFT1 related to cholesterol synthesis were most closely related to GGPS1. GO and KEGG analyses showed that there were certain functions of GGPS1, such as Terpenoid backbone biosynthesis, regulation of small molecule metabolic process, regulation of lipid metabolic process, and cholesterol metabolic process. As the most prominent metabolic alterations in cancer, lipids in the tumor microenvironment play unique roles beyond metabolic requirements that promote cancer progression (Bian et al., 2021; Zhang et al., 2022). Previous research also pointed out that enzymes in the cholesterol synthesis pathway result in pathological cholesterol accumulation, which can be a cancer risk factor (Ershov et al., 2021). Thus, it is speculated that GGPS1 may affect the metabolism of the tumor through some mechanisms.

Outcomes of GSEA analyses showed that a high expression of GGPS1 was associated with the activation of ascorbate and aldarate metabolism, pentose and glucuronate interconversions, porphyrin and chlorophyll metabolism, pathways in cancer, chemokine signaling pathway, oxidative phosphorylation, and PPAR signaling pathway. It seemed that the function of GGPS1 was more relevant to metabolism. Ascorbate and aldarate metabolism and pentose and glucuronate interconversions are significant pathways overrepresented by metabolites to non-fatigued cancer (Feng et al., 2021). Chemokines in cancer often indicated a pro-tumorigenic state (Bule et al., 2021). Exacerbated oxidative phosphorylation dependency frequently characterizes cancer stem cells (Sica et al., 2020). Peroxisome proliferator-activated receptors (PPARs) regulate lipid metabolism and PPAR signaling exerts pleiotropic functions in cancer (Chang and Lai, 2019). Thus, GGPS1 may affect the occurrence and development of tumors by influencing various metabolism and may be beneficial to the occurrence of cancer.

To sum up, the high expression of GGPS1 in OSCC is related to poor prognosis and GGPS1 could be an independent prognostic biomarker, its gene expression level is closely related to the histological stage of cancer. GGPS1 may promote tumorigenesis because of its metabolic function.

While our article presents comprehensive research on GGPS1 expression, survival prognosis of OSCC, gene function prediction, and targeted drugs based on the TCGA database, there are some limitations that should be acknowledged. Firstly, we relied solely on high-throughput transcriptome data from the TCGA database and did not integrate other omics data such as proteomics and genomics. Secondly, we did not conduct any wet-lab experiments on GGPS1 expression in OSCC tumor tissues due to experimental constraints. Future studies should incorporate a broader range of multi-omics data and rigorous preclinical experiments to further validate and expand upon the findings of this study.

## 5 Conclusion

In a word, this study concluded that the high expression of GGPS1 has a significantly unfavorable meaning toward the prognosis of OSCC, and GGPS1 could act as a novel independent biomarker for OSCC.

## Data Availability

The datasets presented in this study can be found in online repositories. The names of the repository/repositories and accession number(s) can be found in the article/Supplementary Material.
